# Hyperinsulinemic Hypoglycemia Diagnosed in Childhood Can Be Monogenic

**DOI:** 10.1210/clinem/dgac604

**Published:** 2022-10-14

**Authors:** Jasmin J Hopkins, Alexandra J Childs, Jayne A L Houghton, Thomas I Hewat, Navoda Atapattu, Matthew B Johnson, Kashyap A Patel, Thomas W Laver, Sarah E Flanagan

**Affiliations:** Institute of Biomedical and Clinical Science, University of Exeter Medical School, Exeter EX2 5DW, UK; Institute of Biomedical and Clinical Science, University of Exeter Medical School, Exeter EX2 5DW, UK; The Genomics Laboratory, Royal Devon University Healthcare NHS foundation Trust, Exeter EX2 5DW, UK; Institute of Biomedical and Clinical Science, University of Exeter Medical School, Exeter EX2 5DW, UK; Paediatric Endocrinology, Lady Ridgeway Hospital, Colombo 00800, Sri Lanka; Institute of Biomedical and Clinical Science, University of Exeter Medical School, Exeter EX2 5DW, UK; Institute of Biomedical and Clinical Science, University of Exeter Medical School, Exeter EX2 5DW, UK; The Genomics Laboratory, Royal Devon University Healthcare NHS foundation Trust, Exeter EX2 5DW, UK; Institute of Biomedical and Clinical Science, University of Exeter Medical School, Exeter EX2 5DW, UK; Institute of Biomedical and Clinical Science, University of Exeter Medical School, Exeter EX2 5DW, UK

**Keywords:** hyperinsulinism, hypoglycemia, genetic testing, monogenic disease, childhood

## Abstract

**Context:**

Congenital hyperinsulinism (HI) is characterized by inappropriate insulin secretion despite low blood glucose. Persistent HI is often monogenic, with the majority of cases diagnosed in infancy. Less is known about the contribution of monogenic forms of disease in those presenting in childhood.

**Objective:**

We investigated the likelihood of finding a genetic cause in childhood-onset HI and explored potential factors leading to later age at presentation of disease.

**Methods:**

We screened known disease-causing genes in 1848 individuals with HI, referred for genetic testing as part of routine clinical care. Individuals were classified as infancy-onset (diagnosed with HI < 12 months of age) or childhood-onset (diagnosed at age 1-16 years). We assessed clinical characteristics and the genotypes of individuals with monogenic HI diagnosed in childhood to gain insights into the later age at diagnosis of HI in these children.

**Results:**

We identified the monogenic cause in 24% (n = 42/173) of the childhood-onset HI cohort; this was significantly lower than the proportion of genetic diagnoses in infancy-onset cases (74.5% [n = 1248/1675], *P* < 0.00001). Most (75%) individuals with genetically confirmed childhood-onset HI were diagnosed before 2.7 years, suggesting these cases represent the tail end of the normal distribution in age at diagnosis. This is supported by the finding that 81% of the variants identified in the childhood-onset cohort were detected in those diagnosed in infancy.

**Conclusion:**

We have shown that monogenic HI is an important cause of hyperinsulinism presenting outside of infancy. Genetic testing should be considered in children with persistent hyperinsulinism, regardless of age at diagnosis.

Congenital hyperinsulinism (HI) is a life-threatening disorder characterized by inappropriate insulin secretion despite low blood glucose (1). Persistent HI can be monogenic, with 38% to 79% (2–5) of individuals found to have a pathogenic variant in one of the known disease-causing genes (6). The variability in these pickup rates will in part reflect how comprehensively the known genes are screened as well as the clinical features of the populations studied.

Biochemical evidence of insulin-induced hypoglycemia is a prerequisite for genetic testing; however, there are no established criteria for the age at diagnosis of HI. This is in contrast to other monogenic disorders of insulin secretion such as neonatal diabetes mellitus and maturity-onset diabetes of the young where the age at presentation of disease is a main criterion for genetic testing (7, 8).

For monogenic HI, hypoglycemia is typically diagnosed in infancy (before 12 months of age) with the most severe cases presenting within the first few days of life (9). In these individuals, disease-causing variants in the KATP channel genes, *ABCC8* and *KCNJ11*, are most common (3–5). Individuals with disease-causing variants in other commonly reported HI genes, such as *GLUD1,* often present later in infancy or, more rarely, in childhood or adulthood (4, 10).

The wide range in age at clinical presentation seen within the same monogenic forms of HI has limited its usefulness in helping to guide genetic testing of this disorder. Furthermore, no studies have systematically screened the known genes in individuals diagnosed outside of infancy, and the large, published cohorts often report limited data on the ages at presentation of HI (3, 4). Together, this makes it difficult to accurately assess how likely monogenic HI is when diagnosed outside of infancy.

In this study, we aimed to investigate the prevalence of monogenic HI in a large cohort of individuals clinically diagnosed with hyperinsulinemic hypoglycemia before 16 years of age. We then compared the pickup rates for a pathogenic variant in those diagnosed before and after 12 months of age and sought to identify genotype-phenotype relationships that could explain the variability in age at presentation of disease within the common genetic subgroups.

## Methods

### Participants

We studied 1848 individuals who had been referred to the Exeter Genomics Laboratory for HI genetic testing. These individuals had all received a clinical diagnosis of HI before the age of 16 years. Insulinoma had not been reported in any individuals. The cohort was divided into 2 groups; those diagnosed with HI in infancy (age < 12 months) and those diagnosed in childhood (age 1-16 years). Clinical information was provided at referral using a standardized request form. Informed consent for genetic testing was obtained from the parents or guardians with assent obtained from all individuals where appropriate. This study was approved by the North Wales Research Ethics Committee (517/WA/0327).

### Molecular Genetic Analysis

Genetic testing was performed in all individuals using DNA extracted from peripheral blood leukocytes. Sanger sequencing of a single gene(s) was performed as a first line test when clinical features were highly predictive of a genetic subtype of HI. For example, *GLUD1* Sanger sequencing was performed in individuals with hyperammonemia or *ABCC8* and *KCNJ11* sequencing in individuals with diazoxide-unresponsive disease, as previously described (11–15).

Targeted next-generation sequencing (tNGS) of 11 HI genes (*ABCC8, CACNA1D, GCK, GLUD1, HADH, HNF1A, HNF4A, INSR, KCNJ11, PMM2,* and *TRMT10A*) was performed in all remaining individuals and those where Sanger sequencing had not identified a disease-causing variant (16). In all samples, copy number status of the targeted genes was assessed using read-depth analysis (16). The coding regions of the *KMT2D* and *KDM6A* genes were also screened in 439 individuals following the introduction of an updated tNGS gene panel to the Exeter Genomics Laboratory. When a disease-causing variant was identified, parental samples were tested when available by Sanger sequencing to establish inheritance.

Sequence data was analyzed to determine if a variant was mosaic. This was performed using the standardized allele ratio in Mutation Surveyor v3.24 software (SoftGenetics, State College, PA, USA) for samples sequenced by Sanger and allele fraction analysis for samples sequenced by tNGS.

### Statistical Analysis

Birth weight Z-scores were calculated using WHO standards, accessed through the Zanthro package in STATA 16 (StataCorp, Texas, USA) (17). A Mann-Whitney test was used to compare the difference in median birth weight Z-scores. The Pearson chi-square test was used to assess the strength of categorical associations.

## Results

**Figure 1.**
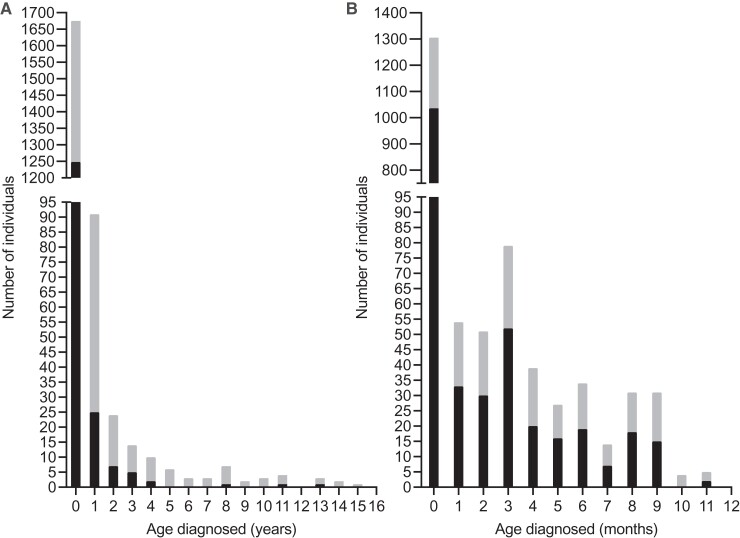
Distribution of age at clinical diagnosis of hyperinsulinism (HI) in probands referred for genetic testing. Gray bars indicate total number of probands, black bars indicate number of probands with a confirmed monogenic etiology. The y-axis is split to include all values. (A) Age at diagnosis of HI in years of the whole cohort (n = 1848). (B) Age at diagnosis of HI in months for those diagnosed in infancy (n = 1675). For those individuals with a genetic diagnosis, 83% (n = 1036/1248) were diagnosed within the first month of life.

Of the 1848 individuals studied, 1675 (90.6%) were clinically diagnosed with HI in infancy (range, birth-51.9 weeks) and 173 (9.4%) were diagnosed with HI in childhood (range, 1-15 years). For the infancy-onset cohort, the median age at diagnosis was 3 days (interquartile range [IQR], 1-21 days), with 77.9% (n = 1305/1675) diagnosed within the first month of life (Fig. 1). For the childhood-onset cohort, the median age at diagnosis was 1.75 years (IQR, 1.17-4 years).

### The Monogenic Pickup Rate Was Lower in Childhood-Diagnosed HI

Disease-causing variants in 13 different genes were identified in 69.8% of the overall cohort (n = 1290/1848). The median age at diagnosis of genetically confirmed HI was 3 days (IQR, 1-14 days) with the eldest child being clinically diagnosed at 13 years (Fig. 1).

**Figure 2.**
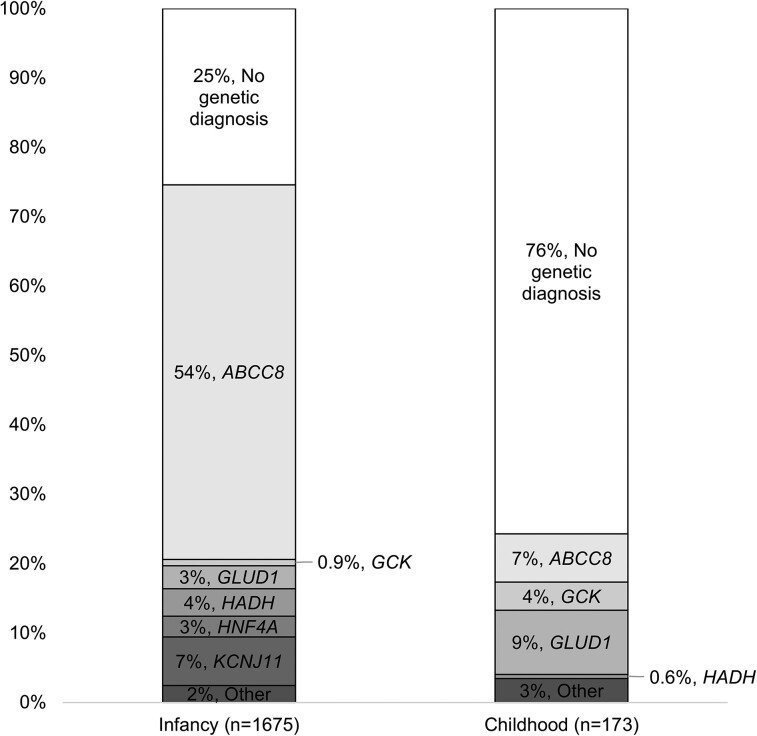
Comparison of genetic causes of hyperinsulinism identified in individuals diagnosed in infancy (< 12 months of age) or in childhood (between 1 and 16 years). Genes with variants in fewer than 1% of patients in the overall cohort were grouped in the “Other” category (*KMT2D, PMM2, KDM6A, HNF1A, INSR, CACNA1D*, and *TRMT10A*).

The proportion of monogenic HI was lower in the childhood-onset cohort compared to the infancy-onset cohort (24.3% [n = 42/173] vs 74.5% [n = 1248/1675], *P* < 0.00001) (Table 1 and Fig. 2). For the childhood-onset monogenic group, the median age at diagnosis was 1.71 years (IQR, 1.02-2.73 years) (Table 1). No significant differences in insulin, blood glucose or birth weight were observed between individuals with or without a genetic diagnosis in the childhood-onset group (Table 2).

**Table 1.** Comparison of features for those with monogenic hyperinsulinism in the infancy-onset (diagnosed < 12 months) and childhood-onset (diagnosed between 1 and 16 years) cohorts

	Infancy-onset cohort	Childhood-onset cohort	*P* value
Proportion monogenic	1248/1675 (74.5%)	42/173 (24.3%)	<0 . 00001
Median age at diagnosis (days)	3 [IQR, 1-7]	624 [IQR, 372-995]	—
Mosaic variants (%)	11 (0.9%)	4 (10%)	<0 . 00001
Dominant *ABCC8* (%) [n]	107 (12%) [907]	2 (17%) [12]	0 . 6
*GLUD1* domain catalytic:allosteric [n]	22:34 [56]	11:5 [16]	0 . 04 *
Median birth weight Z-score [n]	1.4 [1161]	−0.3 [34]	<0 . 00001
Median birth weight Z-score ABCC8 positive [n]	1.5 [843]	−0.5 [9]	0 . 0004
Median birth weight Z-score non-ABCC8-HI [n]	0.2 [136]	−0.1 [25]	0 . 6

Denotes *P* value did not reach statistical significance after adjustment for multiple comparisons.

**Table 2.** Comparison of clinical features provided at referral for individuals with hyperinsulinism diagnosed in childhood (age 1-16 years)

	Monogenic etiology confirmed n = 42	No monogenic etiology identified n = 131	*P* value
**Insulin (pmol/L) [n]**	10.6 [36]	10.7 [111]	1
**Blood glucose (mmol/L) [n]**	2.2 [36]	2.2 [111]	0.9
**Birth weight Z-score [n]**	−0.3 [34]	−0.2 [110]	0.7

*P* values were calculated using Mann-Whitney.

### GLUD1 Was the Most Common Cause of Childhood Monogenic HI

The genetic etiology was different between the 2 cohorts, with *ABCC8*-HI most common in the infancy-onset group (n = 907/1248, 73%) and *GLUD1-*HI most common in those diagnosed in childhood (n = 16/42, 38%) (Fig. 2 and Table 3). Variants in 6 genes (*KCNJ11, HNF4A, KDM6A, HNF1A, INSR,* and *CACNA1D*) were identified only in individuals diagnosed in infancy. *TRMT10A* was the only gene in which a disease-causing variant was identified exclusively in the childhood-onset cohort (Table 3). The homozygous variant was identified in a single proband who had micrognathia and a cleft palate at birth; he was diagnosed with diazoxide-responsive HI (5.2 mg/kg/day) at the age of 3 years and 4 months.

**Table 3.** Number of individuals with hyperinsulinism resulting from disease-causing variants in each gene in the infancy-onset (diagnosed < 12 months) and childhood-onset (diagnosed between 1 and 16 years) cohorts

Gene	Infancy-onset cohort n = 1248	Childhood-onset cohort n = 42	Total n = 1290
*ABCC8*	907 (4 mosaic)	12	919
*KCNJ11*	115	0	115
*GLUD1*	56 (3 mosaic)	16	72
*HADH*	66	1	67
*HNF4A*	48	0	48
*GCK*	15 (1 mosaic)	7 (3 mosaic)	22
*KMT2D*	10 (1 mosaic)	1 (mosaic)	11
*PMM2*	6	4	10
*KDM6A*	10 (2 mosaic)	0	10
*HNF1A*	8	0	8
*INSR*	5	0	5
*CACNA1D*	2	0	2
*TRMT10A*	0	1	1

The number of variants that were found to be mosaic are provided in parentheses.

### Genotype Associations Could Not Fully Explain the Variability in Ages at Diagnosis of HI

Mosaic variants were more common in the childhood-onset cohort than the infancy-onset cohort (10% vs 0.9% (n = 4/42 vs 11/1248), *P <* 0.00001, Table 1). However, 81% (n = 34/42) of variants in the childhood-onset group had been detected in individuals diagnosed with HI in infancy (Table 4), suggesting that the underlying genetics was not the sole factor influencing the age at diagnosis.

**Table 4.** Variants reported as causative in individuals clinically diagnosed with hyperinsulinism in childhood

Gene	Causative variant	Variant identified in an infancy diagnosis	Age at clinical diagnosis, evidence of earlier presentation and known family history
*ABCC8*	p.(Asp29Gly);p.(Ala1493Thr) (c.86A > G;c.4477G > A)	Yes, in reference (18); Yes, in Exeter unpublished cohort This combination of variants has not been reported	3 years
*ABCC8*	p.(Arg74Gln)/p.(Arg74Gln) (c.221G > A/c.221G > A)	Yes, in Exeter unpublished cohort	1.5 years, previous symptoms
*ABCC8*	p.(His125Gln)^‡^/p.? (c.375C > G/c.3992-9G > A)	Yes, in Exeter unpublished cohort/Yes, in Exeter unpublished cohort This combination of variants has not been reported	1.5 years
*ABCC8*	p.(Tyr179Cys)/N (c.536A > G/N)	Yes, in reference (19)	13 years, hypoglycemia detected at age 8
*ABCC8*	p.(Gly228Asp)/N (c.683G > A/N)	Yes, in Exeter unpublished cohort	1 year
*ABCC8*	p.(Arg298Cys)/N (c.892C > T/N)	Yes, in Exeter unpublished cohort	1 year
*ABCC8*	p.(Trp430Ter)/N (c.1290G > A/N)	Yes, in reference (20)	1 year
*ABCC8*	p.(Ala1263Thr)/N (c.3787G > A/N)	Yes, in Exeter unpublished cohort	2.5 years, inherited from affected father
*ABCC8*	p.(Leu1431Phe)^†^/N (c.4291C > T/N)	No	1 year * , inherited from father with diabetes
*ABCC8*	p.(Gly1479Arg)^†^/N (c.4435G > A/N)	Yes, in Exeter unpublished cohort	1.2 years, inherited from mother with gestational diabetes
*ABCC8*	p.?/N (c.1333-1013A > G/N)	Yes, in Exeter unpublished cohort	1.2 years
*ABCC8*	p.?/p.? (c.1817 + 1G > C/c.1817 + 1G > C)	Yes, in Exeter unpublished cohort	1 year *
*GCK*	p.(Trp99Arg)/N (c.295T > C/N)	Yes, in Exeter unpublished cohort	2 years
*GCK*	p.(Val389Leu)/N (c.1165G > C/N)	Yes, in Exeter unpublished cohort	3.6 years * , inherited from father with poor tolerance of fasting
*GCK*	p.(Val389Leu)/N (c.1165G > C/N)	Yes, in Exeter unpublished cohort	3 years
*GCK*	p.(Val389Leu)/N (c.1165G > C/N)	Yes, in Exeter unpublished cohort	11 years
*GCK*	p.(Arg447Leu)/N Mosaic 30% (c.1340G > T/N)	No	1.8 years
*GCK*	p.(Ala454dup)/N Mosaic 17% (c.1361_1363dup/N)	Yes, in Exeter unpublished cohort	3.5 years
*GCK*	p.(Ala454dup)/N Mosaic 30% (c.1361_1363dup/N)	Yes, in Exeter unpublished cohort	1.8 years
*GLUD1*	p.(Arg221Cys)/N (c.820C > T/N)	Yes, in Exeter unpublished cohort	1.8 years
*GLUD1*	p.(Arg221Cys)/N (c.820C > T/N)	Yes, in Exeter unpublished cohort	2.5 years
*GLUD1*	p.(Arg269His)/N (c.965G > A/N)	Yes, in Exeter unpublished cohort	1.1 years
*GLUD1*	p.(Arg269His)/N (c.965G > A/N)	Yes, in Exeter unpublished cohort	1 year
*GLUD1*	p.(Arg269His)/N (c.965G > A/N)	Yes, in Exeter unpublished cohort	1.1 years
*GLUD1*	p.(Arg269His)/N (c.965G > A/N)	Yes, in Exeter unpublished cohort	2 years
*GLUD1*	p.(Arg269His)/N (c.965G > A/N)	Yes, in Exeter unpublished cohort	1.8 years
*GLUD1*	p.(Arg269His)/N (c.965G > A/N)	Yes, in Exeter unpublished cohort	1.7 years
*GLUD1*	p.(Arg269His)/N (c.965G > A/N)	Yes, in Exeter unpublished cohort	4.4 years, inherited from affected mother
*GLUD1*	p.(Glu296Lys)/N (c.1045G > A/N)	No	4 years, inherited from affected mother
*GLUD1*	p.(Glu296Lys)/N (c.1045G > A/N)	No	1 year, hypoglycemic episodes possibly present from 6 months
*GLUD1*	p.(Pro436Leu)/N (c.1466C > T/N)	Yes, in Exeter unpublished cohort	1 year, seizures on day 3 of life *
*GLUD1*	p.(Ile444Met)/N (c.1491A > G/N)	No	8 years, seizures at 1 year
*GLUD1*	p.(Ser445Leu)/N (c.1493C > T/N)	Yes, in Exeter unpublished cohort	1 year, seizures within first days after birth
*GLUD1*	p.(Ser445Leu)/N (c.1493C > T/N)	Yes, in Exeter unpublished cohort	1 year
*GLUD1*	p.(Ala500Thr)/N (c.1498G > A/N)	No	2.08 years, inherited from affected father
*HADH*	p.(Lys260fs)/p.(His207Pro) (c.617delA/c.620A > C)	Yes, in Exeter unpublished cohort/No This combination of variants has not been reported	1 year
*KMT2D*	p.?/N Mosaic 20% Chr11:g.(49415449)_ (49416715)del	No	2.2 years *
*PMM2*	p.?/p.(Phe157Ser) (c.−167G > T/c.470T > C)	Yes, in Exeter unpublished cohort/No This combination of variants has not been reported	1.25 years *
*PMM2*	p.?/p.(Arg141His) (c.−167G > T/c.422G > A)	Yes, in Exeter unpublished cohort	1.5 years
*PMM2*	p.?/p.(Arg141His) (c.−167G > T/c.422G > A)	Yes, in Exeter unpublished cohort	2.8 years *
*PMM2*	p.?/p.(Asp148Asn) (c.−167G > T/c.442G > A)	Yes, in Exeter unpublished cohort/No This combination of variants has not been reported	1.1 years
*TRMT10A*	p.(Gly147fs)/p.(Gly147fs) c.440del/c.440del	No	3.3 years

Protein changes and nucleotide or genomic changes are provided for each variant. Nomenclature is given according to the following transcripts: *ABCC8* (NM_001287174.1), *GCK* (NM_000162.4), *GLUD1* (NM_005271.4), *HADH* (NM_005327.4), *KMT2D* (NM_003482.3), *PMM2* (NM_000303.2) and *TRMT10A* (NM_152292.5).

N: Denotes no pathogenic variant identified on opposite allele. All variants identified were classified as likely pathogenic or pathogenic according to ACMG guidelines (21), unless indicated by^‡^.

Denotes that this individual has been reported previously in the literature. ^†^Denotes a confirmed dominantly acting *ABCC8* variant.

We next investigated the mode of inheritance of *ABCC8*-HI, as dominantly acting variants can cause a milder form of diazoxide-responsive disease compared with recessively inherited *ABCC8* variants (22, 23). There was no difference in the proportion of dominantly acting variants within each group (infancy cohort n = 107/907 vs childhood cohort n = 2/12, *P* = 0.6) (Table 1).

For *GLUD1,* we investigated whether the position of the variant had an impact on the age at diagnosis of HI, due to previous reports that the location of variants impacted the clinical presentation of disease (15, 24). We found that the *GLUD1* variants identified in the childhood-onset cohort clustered in the catalytic domain (exons 6 and 7) of the enzyme (n = 11/16, 69%), while those found in the infancy-onset cohort clustered in the allosteric domain (exons 10, 11, and 12) (n = 34/56, 61%, *P* = 0.04) (Table 1). This association was, however, not significant after correction for multiple comparisons.

### Delayed Diagnosis Was Not the Main Driver of Childhood Diagnoses

**Figure 3.**
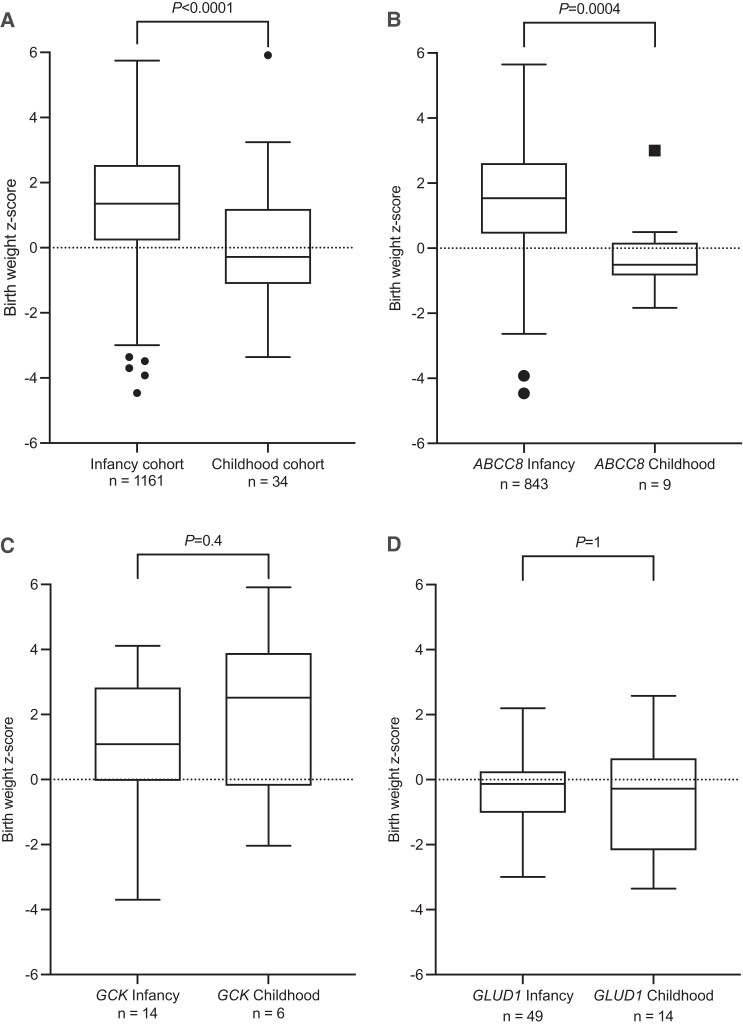
Comparison of birth weight z-score for infancy (< 12 months) and childhood (between 1 and 16 years) diagnosed hyperinsulinism. The middle line depicts the median birth weight Z-score, the length of the box shows the interquartile range, and the whiskers extend to a maximum of 1.5 × IQR from the edge of the box, outliers are plotted individually. *P* values were calculated using Mann-Whitney. Birthweight Z-scores for all genetic causes are shown in panel (A). Data was then separated by gene where there were more than 5 individuals in both the infancy- and childhood-onset cohorts. Data for *ABCC8* is shown in panel (B) *GCK* is shown in panel (C) and *GLUD1* is shown in panel (D).

As we did not detect significant variant-level associations to explain the variability in age at diagnosis, we next investigated whether a delayed diagnosis of HI was likely in those presenting in childhood. Case note review revealed that 5 individuals within the childhood-onset group (9.5%, n = 4/42) had experienced hypoglycemic episodes or seizures during infancy but had not received a clinical diagnosis of HI at that time (Table 4). We next compared corrected birth weights, since birth weight is a surrogate marker for insulin secretion in utero, where it acts as a growth factor. The childhood-onset cohort had a significantly lower median birth weight than the infancy-onset cohort (Z-score: −0.3 vs 1.4, *P* < 0.00001, Table 1 and Fig. 3A). These differences appeared to be driven by *ABCC8*-HI (Z: −0.5 vs 1.5, *P* = 0.0004, Table 1 and Fig. 3B). The median birthweight in the childhood-onset cohort was similar to the population average, providing no evidence of increased insulin secretion in utero. This supports the theory that for the majority of cases, it was not undetected hypoglycemia that resulted in the later age at diagnosis.

## Discussion

We identified pathogenic variants in 69.8% of individuals diagnosed with HI before the age of 16 years. This represents the minimal prevalence of monogenic HI in our cohort, since only the most commonly affected genes were screened. We further showed that HI is monogenic in a significant number of cases with childhood-onset disease, with pathogenic variants in *GLUD1* being the most common etiology.

The pickup rate was lower but still substantial in childhood-diagnosed HI, with the monogenic cause identified in 74.5% of individuals diagnosed in infancy compared to 24.3% of individuals diagnosed in childhood. The age at diagnosis of HI in our genetically solved childhood-onset cohort ranged from 1 to 13 years, with 75% diagnosed between ages 1 and 2.7 years (Fig. 1 and Table 1). Although previous studies have separated individuals based on age at diagnosis of HI (25), this is the first study to report the prevalence of known variants within these age groups.

Some differences in the genetic subtypes of HI were observed between the infancy and childhood groups, with disease-causing variants in 6 genes identified exclusively within the infancy-onset cohort. Although it is possible that our childhood-onset cohort was underpowered to detect these rare forms of HI, we were also unable to find reports of childhood-onset cases in the literature. This suggests that variants in these genes predominantly cause early-onset HI. The only gene in which a variant was found exclusively in the childhood-onset cohort was *TRMT10A*. This represents the second reported case of a disease-causing *TRMT10A* variant in a child referred for HI genetic testing (26).

We found that mosaic variants were more common in the childhood-onset than the infancy-onset cohort (*P* < 0.00001). These variants were detected from blood samples, and we were unable to test pancreatic DNA. It seems likely that in these individuals there is a mixed population of cells within the pancreas, in which some cells do not harbor the disease-causing variant, which impacts the severity and age at presentation of HI. No further significant associations between the genotype and age at presentation of disease were identified.

To assess whether a delayed clinical diagnosis could explain the variability in age at presentation of HI, we searched clinical request forms for evidence of unexplained hypoglycemia in infancy. This identified 4 individuals clinically diagnosed with HI in childhood with potential previous symptoms of hypoglycemia, including seizures. To investigate whether a delayed diagnosis was common, we studied the corrected birth weights of our cohort and found no evidence to support the presence of increased insulin secretion in utero and thus undiagnosed HI from birth in the childhood-onset cohort. In fact, we found that the birth weights of individuals diagnosed with *ABCC8*-HI in childhood were significantly lower than those diagnosed in infancy, in keeping with these being true cases of later-onset disease rather than infancy-onset cases with a delayed diagnosis.

Taken together, the lower birth weights and the finding that 75% of individuals in the childhood-onset cohort were diagnosed before 2.7 years suggest that these monogenic cases represent the tail end of the normal distribution in age at diagnosis. This is supported by the finding that 81% of the variants identified in the childhood-onset cohort were detected in those diagnosed in infancy, suggesting that alternative genetic or environmental factors are having an impact on the age at presentation of HI in these individuals.

Our study has limitations. First, individuals were ascertained based on clinical referral. To determine the true prevalence of monogenic HI, a systematic screen is required. Second, while we screened the most common causes of HI (accounting for 69.8% of cases) we did not screen all known genetic causes of HI. We may therefore have missed pathogenic variants in the genes reported to cause syndromic HI; however, this would only have a small impact since they are expected to be rare (27). Insulinomas are a well-recognized cause of HI, with the youngest reported age at diagnosis being 2 years (28); while evidence of this as an underlying cause led to exclusion, they were not systematically screened for in our cohort. We compared individuals diagnosed with HI in childhood, defined as age 1 to 16 years, to those diagnosed in infancy, younger than 12 months. While these age categories have been used in previous studies (25), alternative cutoffs may have yielded different results. Finally, although we studied a large overall cohort, the sample size for the childhood cohort was relatively small and may not be truly representative.

In conclusion, we have shown that a significant number of individuals diagnosed with HI in childhood can have a monogenic etiology. Although the likelihood of identifying a pathogenic variant was lower for childhood-onset than infancy-onset HI, the number of children receiving a genetic diagnosis in our cohort was substantial and therefore genetic testing should be considered in all cases with persistent HI, regardless of age at diagnosis.

## Data Availability

Restrictions apply to the availability of some or all data generated or analyzed during this study to preserve patient confidentiality or because they were used under license. The corresponding author will on request detail the restrictions and any conditions under which access to some data may be provided.

## Abbreviations

HI: hyperinsulinism
IQR: interquartile range
tNGS: targeted next-generation sequencing
